# Mechanical thrombectomy combined with recombinant tissue plasminogen activator thrombolysis in the venous sinus for the treatment of severe cerebral venous sinus thrombosis

**DOI:** 10.3892/etm.2015.2198

**Published:** 2015-01-21

**Authors:** YONG ZHEN, NAN ZHANG, LIANG HE, LINHAI SHEN, KAIXUAN YAN

**Affiliations:** Department of Neurosurgery, Northern Jiangsu People’s Hospital, Yangzhou, Jiangsu 225001, P.R. China

**Keywords:** cerebral sinus thrombosis, recombinant tissue plasminogen activator, endovascular interventional therapy, thrombolytic therapy

## Abstract

The aim of the present study was to assess the effectiveness and safety of endovascular interventional therapy, which is mechanical clot disruption combined with intrasinus thrombolytic therapy with recombinant tissue plasminogen activator (rt-PA), for severe cerebral venous sinus thrombosis (CVST). The records of eight patients with CVST confirmed by computed tomography, magnetic resonance imaging (MRI), magnetic resonance venography (MRV) and/or digital subtraction angiography were analyzed. Of the eight cases, the Glasgow Coma Scale (GCS) scores were between 4 and 9 with a mean value of 8.3±2.7. All the patients had venous infarction and two cases had intracranial hemorrhagic infarcts. Mechanical clot destruction combined with intrasinus thrombolytic therapy with rt-PA was performed under general anesthesia. Intravenous heparin therapy and intracerebral pressure control were applied during this period. One patient succumbed and the other seven patients showed good treatment efficacy. The GCS scores of the seven patients reverted to 15 upon discharge from the Northern Jiangsu People’s Hospital (Yangzhou, China). With regard to the modified Rankin score of the seven patients three months following surgery, six patients scored 0 and one patient scored 1. MRI and MRV follow-up examinations were performed for 3–15 months. Complete recanalization of the criminal sinus, which refer to the sinus attributable to the infarction or hemorrhage, was observed in five cases and partial recanalization was observed in two cases. Symptoms were monitored for 3–24 months and no recurrence was observed. Therefore, mechanical thrombectomy combined with intrasinus thrombolytic therapy with rt-PA is safe and effective for patients with severe CVST.

## Introduction

The incidence of cerebral venous sinus thrombosis (CVST) is 3–4/million/year. CVST accounts for <1% of apoplexy. Misdiagnosis and missed diagnosis of CVST are common due to the atypical symptoms of the condition at an early stage (1–3). However, the extensive use of new imaging techniques, including computed tomography (CT), computed tomography venography, magnetic resonance imaging (MRI) and magnetic resonance venography (MRV), allows for the early diagnosis of CVST. The present first-line treatment is systemic anticoagulation (4,5). Although early diagnosis and active anticoagulant therapy has reduced the mortality of CVST from 40% in the 1980s to the current rate of 4.39–13% (6–8), the disability and mortality rates in patients with acute onset CVST remain relatively high despite the use of heparin therapy. In the International Study on Cerebral Vein and Dural Sinus Thrombosis, Ferro *et al* reported that the mortality rate of patients in a coma was 38% (7). Active treatment should be provided to these patients. Evidence from minor cases indicates that local thrombolytic therapy is relatively safe and effective in rapidly recanalizing thrombosed sinuses and reversing neurological deficits (9,10). Local intrasinus thrombolysis dissolves the thrombus by infusing the thrombolytic drug into the occluded sinus and employing mechanical thrombectomy to recanalize the sinus as early as possible. Mechanical thrombectomy may strengthen the thrombolytic effect of drugs. As a common type of thrombolytic agent, recombinant tissue plasminogen activator (rt-PA) is widely utilized in intra-arterial thrombolysis and is safer compared with urokinase. Therefore, in the present study, intrasinus mechanical thrombectomy was combined with thrombolysis using rt-PA to manage severe CVST. However, due to the absence of randomized controlled trials, the exact role of intrasinus thrombolysis and mechanical thrombectomy in the management of CVST is unclear, particularly with regard to patient selection, optimal time to intervene and contraindications of this therapy. Retrospective data analysis of eight patients with severe CVST who were subjected to local thrombectomy combined with intrasinus rt-PA thrombolysis was conducted to evaluate the effectiveness and safety of this endovascular interventional therapy for severe CVST.

## Materials and methods

### General materials

Eight patients with CVST were treated in Northern Jiangsu People’s Hospital (Yangzhou, China) between December 2009 and December 2011. Of the eight patients, two were males and six were females. Patient age ranged between 19 and 48 years with an average of 27.5±10.4 years. One patient was in puerperium, two patients had a long-term history of contraceptive use, one patient had a history of deep vein thrombosis and two patients had severe anemia, the remaining two patients had no evident risk factors. The clinical manifestations were as follows: Headache associated with progressive disturbance of consciousness in all the patients, focal neurological deficit symptoms in six patients and epilepsy in two patients. Two patients progressed to the stage of cerebral hernia while undergoing endovascular treatment. Glasgow Coma Scale (GCS) scores were between 4 and 9 with an average score of 8.3±2.7 points. The duration from the onset of symptoms to the receipt of interventional therapy ranged between 3 and 12 days. The study was conducted in accordance with the Declaration of Helsinki and was approved by the Ethics Committee of Northern Jiangsu People’s Hospital. Written informed consent was obtained from all the participants or their families.

### Imaging materials

Patients were diagnosed via CT, MRI, MRV and/or digital subtraction angiography (DSA). Thrombus locations were as follows: Superior sagittal sinus in one case, superior sagittal sinus and left transverse sinus in one case, superior sagittal sinus and straight sinus in one case, straight sinus and left transverse sinus in four cases and straight sinus and right transverse sinus in one case. The patients exhibited varied degrees of cerebral venous infarction and two cases exhibited cerebral hemorrhage.

### Treatment method

Transforaminal herniation occurred in one patient 3-h after hospitalization and decompressive craniectomy was performed. The other seven patients were subjected to systemic anticoagulation with heparin following definite diagnosis, however, progressive aggravation or no improvement of symptoms was observed within 24 h. Interventional treatment was provided with general anesthesia. Vessel sheaths were inserted in the left femoral vein and right femoral artery. Arterial catheterization with a 5 F catheter was performed in the cerebral artery. The procedure confirmed the location of thrombosis and was utilized for the arterial administration of thrombolytic drugs in addition to the evaluation of the sinus thrombolytic effect. A 6 F Envoy support catheter (Cordis Neurovascular, Inc., Miami Lakes, FL, USA) was inserted through the femoral vein. The support catheter was placed in the left or right sigmoid sinus with the aid of a 0.35 loach guidewire based on the location of the lesion. A Prowler 14 microcatheter (Cordis Neurovascular, Inc.) guided by an Essence 14 micro guidewire (Cordis Neurovascular, Inc.) penetrated the thrombosis. If the thrombus was in the superior sagittal sinus or transverse sinus, the Trensend 300 Floppy exchange guidewire (Boston Scientific Target, Bayside Parkway, Fremone, CA, USA) was selected and placed in the dilated sacculus after being expanded in advance. By contrast, if the thrombus was in the straight sinus, a microcatheter, with which the rt-PA (Actilyse; BoehringerIngelheim Pharmaceuticals, Ingelheim, Germany) was administered, was directly placed in the distal end of the thrombus. Following thrombolysis, the support catheter was removed, whereas the microcatheter remained. The arterial catheter and sheath were removed. Patients were administered 15 mg rt-PA via the microcatheter and 5 mg rt-PA via the arterial catheter. Heparinization was continued and the activated partial thromboplastin time (APTT) was controlled to twice that of the normal level following surgery. Three to five days after surgery, 20 mg rt-PA was administered daily via the microcatheter. The microcatheter and vessel sheath were removed when the symptoms improved. When the patients were conscious, warfarin was administered sequentially for one year to adjust the international normalized ratio between 2.0 and 3.0 and the provision of heparin was discontinued.

## Results

### Patients

One patient succumbed to transforaminal herniation that occurred prior to thrombolysis. Although spontaneous breathing resumed following decompressive craniectomy, the bilateral pupils dilated during thrombolysis and surgery was terminated. The other seven patients recovered well. Consciousness recovered between two and five days after surgery and muscle strength recovered between six and ten days following surgery. One patient remained afflicted with an affective disorder, one patient developed mild diplopia and one patient developed a fine movement disorder of the upper limbs upon discharge from the hospital. The modified Rankin Scale scores improved three months following surgery with six patients having a score of 0 and one patient having a score of 1. Reexaminations with MRI and MRV occurred for 3–15 months. The sinuses of five patients were completely recanalized and the sinuses of two patients were partially recanalized. Follow-up lasted for 3–24 months and no recurrence was observed.

### Typical case 1 (female, 47 years-old)

**Figure 1:**
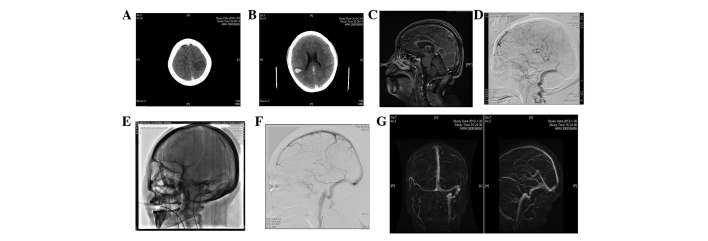
Catheter-directed thrombolysis in the superior sagittal sinus. (A) Intracranial left parietal lobe infarction shown by CT; (B) hemorrhage at the junction of the right temporal and occipital lobes; (C) thrombosis of the superior sagittal sinus and straight sinus shown by enhanced MRI; (D) DSA showed that the superior sagittal sinus and straight sinus did not develop; (E) sacculus expanded during surgery; (F) on day 11 following surgery, review with DSA showed that the superior sagittal sinus, straight sinus and left transverse sinus developed well; (G) one year later, review with MRV showed that the superior sagittal sinus, straight sinus and left transverse sinus developed well. CT, computed tomography; MRI, magnetic resonance imaging; DSA, digital subtraction angiography; MRV, magnetic resonance venography.

Sudden headache and vomiting occurred 2 days prior to hospitalization. Heparinization was performed following admission. However, the disease was progressively aggravated. Minor epilepsy was observed three times and the GCS score was 9. CT examination revealed that the left parietal lobe was infarcted and hemorrhage was identified at the junction of the right temporal and occipital lobes (Fig. 1A and B). MRI revealed that thrombi existed in the superior sagittal sinus and straight sinus (Fig. 1C). In addition, DSA showed that the superior sagittal sinus and straight sinus was not phanerous due to thrombosis or congenital dysplasia. The cortical veins were connected to the sigmoid sinus through the sylvian vein, cavernous sinus, pterygoid plexus and vein of Labbé (Fig. 1D). The sacculus expanded during surgery (Fig. 1E). The microcatheter was left in the superior sagittal sinus for 3 days after surgery. On day 2 following surgery, the awareness of the patient improved significantly, simple verbal exchanges became possible and right side muscle strength was at level 3, according to the Oxford Scale. On day 8 following surgery, the patient was conscious, speech was fluent and muscle strength was at level 5. DSA revealed that the superior sagittal sinus, straight sinus and left transverse sinus were well-developed at day 11 following surgery (Fig. 1F). After one year, the patient exhibited no neurological deficit and MRV demonstrated that the superior sagittal sinus and straight sinus had recovered well (Fig. 1G).

### Typical case 2 (female, 21 years-old)

**Figure 2:**
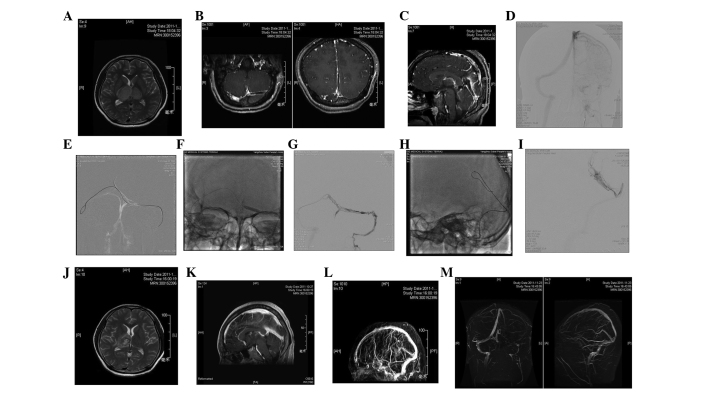
Catheter-directed thrombolysis in the straight sinus. (A) Head MRI T2 weighted image showed that the bilateral thalami were infarcted and the right side was more severe; (B) thrombus in the straight sinus as shown by enhanced MRI; (C) thrombus in the left transverse sinus and superior sagittal sinus as shown by enhanced MRI; (D) DSA showed that the left transverse sinus and straight sinus did not develop; (E) guidewire entered the transverse sinus during surgery; (F) sacculus expanded during surgery; (G) left transverse sinus was open during surgery; (H) microcatheter was placed in the straight sinus; (I) microcatheter angiography revealed that the straight sinus developed following thrombolysis; (J) 2 weeks after surgery, review of the T2 weighted image showed that disease extent of the thalamus had reduced; (K) enhanced imaging showed that the superior sagittal sinus and straight sinus were open; (L) MRV showed that the superior sagittal sinus and straight sinus were open; (M) 6 weeks after surgery, MRV showed that the straight sinus was open but the left transverse sinus was closed. CT, computed tomography; MRI, magnetic resonance imaging; DSA, digital subtraction angiography; MRV, magnetic resonance venography.

Headache with left limb weakness lasted for 5 days and unconsciousness lasted for 1 day prior to hospitalization. Physical examination revealed the GCS score to be 7. The patient had aphonia and both eyes gazed to the right side. The left nasolabial fold became shallow and MRI revealed that the bilateral thalami were infarcted, particularly on the right side (Fig. 2A). Enhanced imaging showed that thrombi existed in the straight sinus, left transverse sinus and superior sagittal sinus (Fig. 2B and C). DSA revealed that the left transverse sinus and straight sinus did not develop (Fig. 2D). The microcatheter was placed in the straight sinus via the left transverse sinus and the sacculus expanded during surgery (Fig. 2E–H). Awareness improved at day 2 following surgery, but the patient developed hypersomnia and aphasia. At day 3 after surgery, the patient regained consciousness and was able to have simple verbal exchanges. Left side muscle strength was at level 3 and right side muscle strength was at level 4. At day 9 following surgery, the patient’s speech was fluent, right side muscle strength was at level 5 and left side strength was at level 4+. MRI and MRV examinations at week 2 following surgery revealed that the disease was in remission and the superior sagittal sinus and straight sinus had recanalized (Fig. 2I–K). At week 6 after surgery, the patient’s muscle strength recovered and MRV showed that the straight sinus had recanalized, whereas the left transverse sinus was closed (Fig. 2L and M).

## Discussion

Although the prognosis of CVST has improved significantly through the administration of heparin, certain patients with CVST continue to show poor treatment effects. An appropriate treatment approach at the right time remains to be established.

Ideal treatment should aim to prevent further formation of thrombi, recanalize the venous sinus and establish collateral circulation. In the present study, symptomatic treatment, heparinization, thrombolysis and interventional means were employed. Heparin therapy was provided once intracranial venous sinus thrombosis had been diagnosed. The efficacy and safety of unfractionated heparin or low molecular weight heparin for CVST treatment have already been confirmed by a number of studies and has been shown to not increase the risk of rebleeding for CVST with intracranial hemorrhage (3,5,11–15). The dosage of heparin, which may control the APTT level to twice that of the normal level, was appropriate. However, antiepileptic, reduction of intracranial pressure and symptomatic supportive treatment should also be provided. A previous study of 624 patients demonstrated that although anticoagulant therapy was provided, 21% of the patients exhibited a poor prognosis and the mortality rate was 8.2%. In addition, the coma patients exhibited a mortality rate of 38% (7). Therefore, if the effect of heparin treatment is not evident or if symptoms become aggravated, more active treatment should be considered, particularly for patients with severe CVST in the following four situations: i) intracranial hematoma; ii) coma; iii) mental symptoms; and iv) intracranial deep vein thrombosis (1). Deep vein thrombosis often leads to rapid deterioration in patient condition. In the present study, there were six patients whose thrombus involved the straight sinus and basal vein. Patients with severe CVST required active surgical intervention. Based on the results of the present study, we hypothesized that a more active therapeutic approach should be applied when consciousness disturbance worsens in the first 24-h after the adoption of intravenous heparinization.

At present, common treatment methods include intravenous thrombolysis, arterial thrombolysis, local sinus thrombolysis via a microcatheter, local mechanical thrombectomy in the venous sinus and sinus stent or a combination of the aforementioned methods (16–20). A decompressive craniectomy should be performed on patients with severe high intracranial pressure and cerebral hernia (21–23). In the present study, these patients were treated mainly by local thrombectomy combined with local rt-PA thrombolysis in the sinus via a microcatheter. The microcatheters were left for continuous local thrombolysis for 3–5 days after surgery. In the seven patients that survived, the venous sinuses of five patients were completely recanalized and those of the two patients that were partially recanalized, the symptoms improved. The results revealed that the lesion of severe patients involved multiple venous sinuses and that treatment should be combined with CT and MRI results (3,24). The criminal sinus was first analyzed and mechanical thrombectomy was then performed. A micro guidewire was passed through the thombosis repeatedly to mechanically loosen the thrombosis and the sacculus was employed to expand the thrombus. Microcatheters were then placed in the thrombus and were pulled back from the distal end during thrombolysis. Finally, the microcatheters were placed in the criminal sinus for long-term thrombolysis. Complete recanalization was not required during surgery due to the long formation time of venous sinus thrombosis. Heparinization with continuous local thrombolysis may continue to recanalize the sinus gradually provided that a blood flow exists in the venous sinus.

Therefore, local thrombectomy combined with local rt-PA thrombolysis in the sinus via a microcatheter, administered as early as possible, is a safe and effective method of treatment for patients with severe CVST. However, this conclusion requires the support of random double-blind controlled trials with a large sample size.
